# Structural brain network topological alterations in stuttering adults

**DOI:** 10.1093/braincomms/fcac058

**Published:** 2022-03-10

**Authors:** Vincent L. Gracco, Anastasia G. Sares, Nabin Koirala

**Affiliations:** 1 Haskins Laboratories, New Haven, CT, USA; 2 School of Communication Sciences & Disorders, McGill University, Montreal, Canada; 3 Department of Psychology, Concordia University, Montreal, Canada

**Keywords:** stuttering, community structures, network-based statistics, controllability, diffusion-weighted imaging

## Abstract

Persistent developmental stuttering is a speech disorder that primarily affects normal speech fluency but encompasses a complex set of symptoms ranging from reduced sensorimotor integration to socioemotional challenges. Here, we investigated the whole-brain structural connectome and its topological alterations in adults who stutter. Diffusion-weighted imaging data of 33 subjects (13 adults who stutter and 20 fluent speakers) were obtained along with a stuttering severity evaluation. The structural brain network properties were analysed using network-based statistics and graph theoretical measures particularly focussing on community structure, network hubs and controllability. Bayesian power estimation was used to assess the reliability of the structural connectivity differences by examining the effect size. The analysis revealed reliable and wide-spread decreases in connectivity for adults who stutter in regions associated with sensorimotor, cognitive, emotional and memory-related functions. The community detection algorithms revealed different subnetworks for fluent speakers and adults who stutter, indicating considerable network adaptation in adults who stutter. Average and modal controllability differed between groups in a subnetwork encompassing frontal brain regions and parts of the basal ganglia. The results revealed extensive structural network alterations and substantial adaptation in neural architecture in adults who stutter well beyond the sensorimotor network. These findings highlight the impact of the neurodevelopmental effects of persistent stuttering on neural organization and the importance of examining the full structural connectome and the network alterations that underscore the behavioural phenotype.

## Teaser

Persistent developmental stuttering is a speech motor disorder that primarily affects normal speech fluency but encompasses a complex set of behavioural symptoms. Here, we investigated the whole-brain structural connectome and its topological alterations in adults who stutter and identified extensive structural network differences in neural architecture well beyond the sensorimotor network.

## Graphical Abstract

**Graphical Abstract:**
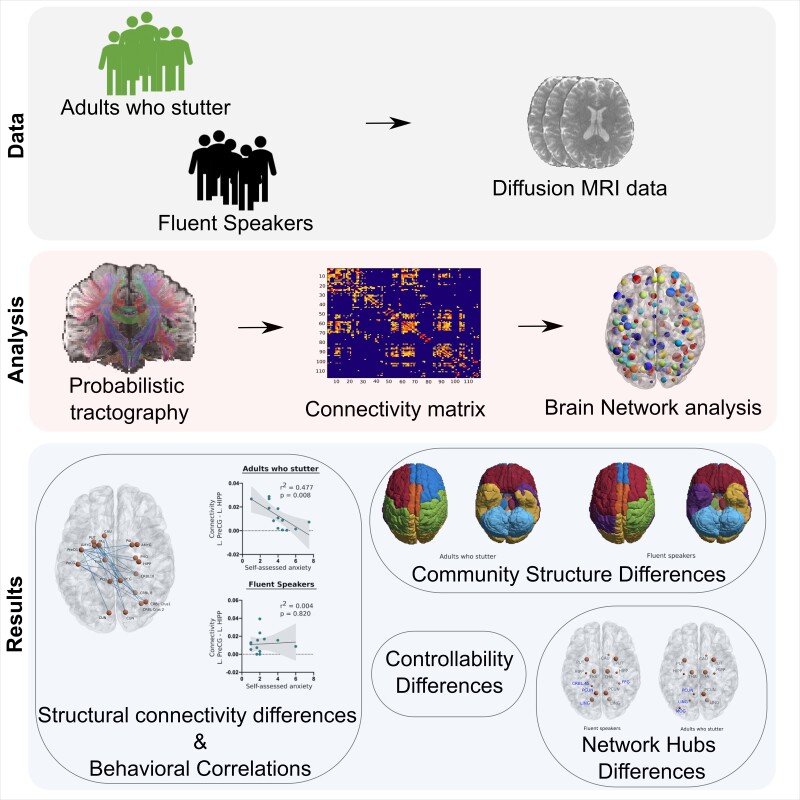

## Introduction

Persistent developmental stuttering, also known as childhood onset fluency disorder, is primarily a problem with the fluent production of speech but also encompasses a complex set of symptoms ranging from problems in sensorimotor integration to socioemotional challenges. Compared with non-stuttering fluent speakers (FS), adults who stutter (AWS) tend to show a range of functional and structural brain differences that have been the focus of much neuroimaging work. Studies involving functional imaging have identified *over-activation* in dominantly right hemispheric motor areas (primary motor cortex, premotor cortex, supplementary motor area and inferior frontal gyrus), the cerebellum (CBM) and reduced *activation* of left inferior frontal gyrus and superior and middle temporal gyri.^1–8^ Structurally, stuttering is associated with reduced grey and white matter volumes^9^ and disrupted white matter organization in left central operculum, bilateral inferior frontal, peri-Rolandic, inferior frontal, subcortical regions and in deep white matter tracts.^10–14^ In addition, abnormal lateralization for speech production has been reported^15–17^ with reduced structural asymmetry^18,19^ and inadequate cortical thinning in Broca's area with age.^20^ Recent studies of AWS examining white matter connectivity using diffusion data have found reduced connectivity strength in a number of areas with a predominance in the left hemisphere.^10,21^ The corticocortical connectivity of AWS is generally weaker in bilateral brain areas associated with speech production including parts of the left peri-Rolandic sensorimotor and premotor cortex, most notably the left ventral premotor cortex and middle primary motor cortex. Overall, the majority of neuroimaging studies of AWS have focussed on brain areas directly associated with the sensorimotor process of speaking^22,23^, even though verbal communication is dependent on and reflects the interaction of cognitive and emotional processes.^24,25^

Individuals who stutter experience feelings of fear of humiliation, embarrassment and negative evaluation in social or performance-based situations which contribute to the development of a high rate of social anxiety disorder.^26,27^ The negative consequences of stuttering can begin early, with evidence of pre-school children who stutter experiencing bullying, teasing, exclusion and negative peer reactions.^28–30^ These consequences are intensified during the school years when children become more involved in social and speaking situations and continue into adulthood, with negative effects on quality of life and socioemotional experiences.^31,32^ AWS associates the moment of stuttering with a sensation of anticipation and loss of control, which leads to behavioural reactions that become deeply ingrained over time.^32^ These aversive communicative events become generalized and associated with a breakdown in speech fluency. In this context, it is important to recognize that persistent stuttering is first and foremost a neurodevelopmental disorder,^33^ and its cognitive, social and emotional consequences are likely secondary, stemming from the difficulty that these individuals have coordinating their speech.

The precise neurodevelopmental origin of stuttering is currently unknown, complicated by the fact that it is only observable after the onset of speech. Epidemiological studies reflect a range of potential causal factors that reflect genetic, epigenetic and environmental interactions. One of the striking consequences of neurodevelopmental disorders, in general, is a complex ontogenetic adaptation of brain networks due to alterations in neurogenesis, cell migration and neuronal connectivity.^34–36^ Using a combined analysis of functional connectivity MRI data and gene expression maps from both children and AWS, a potential causal link between gene mutations and aberrant brain connectivity in stuttering has been proposed, having to do with lysosomal dysfunction.^37^ For AWS, additional adaptations impacting brain structure and function include environmental factors such as socioeconomic status, impaired child–parent interactions, conflicts with parents, negative parental reactions to normal childhood dysfluency and other socioemotional developmental consequences leading to emotional reactivity, social anxiety^26,38–41^ and problems with executive function, learning, memory and emotional (self)-regulation.^25,42–44^ While empirical data clearly suggest a complex neurodevelopmental disorder that extends beyond the overt symptoms of dysfluency,^33,45–47^ there has not been an extensive and comprehensive evaluation of the structural networks in AWS. Most of the neuroimaging work evaluating brain structure has focussed on assessing connectomic differences in brain areas directly associated with the speech production and speech motor planning^10,11^. In the current study, we chose to examine the full spectrum of brain regions in a cohort of AWS and FS using network and graph theoretical analyses^48–50^ to assess network connectivity, organization and integrity. The expectation is that genetic/neurodevelopmental differences,^37^ in combination with environmental/risk factors, have resulted in structural connectivity changes that impact the organization and interaction amongst brain regions that extend well beyond the speech motor network. These structural alterations contribute to network-level changes that impact the speech motor network, overall network communication and processing efficiency across multiple behavioural domains.

## Methods

### Data acquisition

A total of 33 subjects were analysed including 13 adults (5 males, 8 females) who stutter (AWS: mean age: 28.77 ± 11.11 years) and 20 FS (10 males, 10 females) (FS: mean age: 29.6 ± 10.41 years). The subjects, also included in our previous studies,^51,52^ had no known neurological, speech or language problems besides stuttering and FS having no history of stuttering. This study was approved by the McGill Faculty of Medicine Institutional Review Board and the Yale Institutional Review Board in accordance with principles expressed in the Declaration of Helsinki; informed written consent was obtained from participants prior to their involvement in the project.

For all subjects, whole-brain high-resolution T_1_-anatomical image and diffusion-weighted images (DWI) were obtained using 3T MR-scanner (Siemens TrioTim). The T_1_-anatomical image was obtained using an magnetization-prepared rapid acquisition with gradient echo (MPRAGE) sequence with repetition time (TR) = 2300 ms, echo time (TE) = 2.98 ms, flip angle = 9° and field of view (FoV) = 256 mm. DWI was obtained using a single-shot spin-echo diffusion-weighted echo-planar imaging sequence of 2 mm isometric voxel resolution covering an FoV of 244 mm × 244 mm, matrix size of 122 × 122, TR = 8800 ms, TE = 87 ms and slice thickness of 2 mm. DWI was acquired along 60 non-collinear gradient directions with a *b*-value of 1000 s/mm^2^ along with 9 reference volumes with *b* = 0 s/mm^2^ (no diffusion weighting) for each acquisition.

All subjects underwent a speech evaluation before the MRI data acquisition, details of which are described in our previous studies.^53,54^ In brief, a trained speech–language pathologist specializing in stuttering, blinded to each participant's classification, was given 10-min videos of natural speech productions from the testing session and was asked to classify them along with rating the severity of each stuttering participant according to the Stuttering Severity Instrument, fourth edition (SSI-4). In addition, every stuttering participant self-rated their stuttering severity and speaking anxiety on a scale of 1–9 with 1 corresponding to ‘no stuttering/anxiety’ and 9 being ‘very severe stuttering/anxiety’ reflecting their experience with speech in daily life. FS also rated their anxiety about speaking generally. The details of the demographics are presented in Table 1.

**Table 1:** Demographics of the stuttering subjects included in the study

Subject	Age (years)	Sex	SSI-4 score	Self-rated severity	Self-rated anxiety
1	24	Female	23	5	3
2	22	Female	13	3.5	4.75
3	28	Female	29	7	3
4	40	Female	17	4	2.5
5	23	Female	32	5	3.5
6	18	Female	10	2	7
7	31	Male	13	3.5	5
8	23	Male	25	4	4
9	27	Female	8	3.33	4
10	51	Male	26	7.5	6
11	49	Male	13	3	5
12	20	Female	14	4	4.5
13	18	Male	22	4.5	4.5

Here, SSI-4 is the Stuttering Severity Instrument, fourth edition used by the speech–language pathologist for classifying stuttering in terms of severity. For self-rated stuttering severity and speaking anxiety, a scale of 1–9 was used with 1 corresponding to ‘no stuttering/anxiety’ and 9 to ‘very severe stuttering/anxiety’.

### Data processing

#### Probabilistic tractography

The obtained DWI from all subjects were controlled for data quality^55,56^ and pre-processed using inbuilt functionality in FMRIB software library (FSL) (version 6.0.1) described in detail elsewhere.^57–59^ In brief, susceptibility and motion artefact correction and diffusion tensor modelling were performed using the diffusion toolbox (FDT, part of FSL). Crossing fibres’ distribution was estimated using BEDPOSTX (implemented in FSL) and the probability of major and secondary fibre directions was calculated. All images were aligned and affine transformed into the stereotactic Montreal Neurosciences Institute (MNI)-152 space. A multi-fibre model was fit to the diffusion data at each voxel, allowing for tracing of fibres through regions of crossing or complexity. Here, we drew 5000 streamline samples from each seed voxel to form an estimate of the probability distribution of connections from each seed voxel. The obtained probabilistic distribution was used to build the connectivity matrix. A connectivity matrix was obtained using the seed masks for 116 regions of interest (ROIs) each as a node defined by the Automated Anatomical Labelling atlas^60^ for each subject. The links or entries in the connectivity matrix represent the ratio of the number of samples (or streamlines) that pass through an ROI to all generated streamlines from an ROI or the probability of the connection that exist between any two regions.

#### Network-based statistic

Network-based statistic (NBS) analysis was applied to assess differences in the interregional connectivity matrices between the groups. NBS analysis deals with the multiple comparisons problem posed by connectomic data by evaluating the null hypothesis at the level of interconnected subnetworks rather than individual connections.^61,62^ Here, the connectivity matrices obtained from probabilistic tractography were subjected to NBS analysis to identify the difference between the groups. Two thresholds of the *t*-statistic >1.7 (*P* = 0.047) and >2 (*P* = 0.02) were selected for showing two different networks with different number of connections and levels of statistical significance (*P* < 0.05). Five thousand permutations were generated shuffling the participant labels to build the null distribution.^63^ Note that the choice of threshold only affects the sensitivity of the method and still guarantees the significance and control for family wise error rate. Finally, the networks showing impaired connectivity between regions compared with FS were reported.

#### Bayesian power analysis

Given our small sample size, we assessed whether the structural connectivity differences were reliable by examining the effect size with Bayesian power analyses computed using the freely available software Bayesian estimation^64,65^ in R. All the connections showing significant difference in the NBS analysis were used as inputs for both groups *y*_1_ (FS) and *y*_2_ (AWS) to test for group differences using the Bayesian posterior distribution analyses. The Markov Chain Monte Carlo approach was used to compute Bayes factor for the choice of priors with the simulation of 100 000 sampling steps.^66,67^

#### Behavioural correlations

The relationship of the connectivity difference to the behavioural measures was assessed by computing the Pearson correlation coefficient for both groups [AWS: *n* = 13, FS: *n* = 14 (complete behavioural measures were only available for 14 of 20 FS), controlled for age and sex] for the ratings of stuttering severity and self-rated anxiety scores from the AWS.

#### Community structure

Modules or network communities are defined as clusters of nodes derived from a decomposition of the network into subcomponents. These subcomponents have strong internal coupling, but weak external interrelation.^68,69^ Based on the topology of the network, these modules were detected in a purely data-driven way with each node assigned to each module to assess network function.^70^ Two different methods to compute the modules based on assortative and disassortative models were implemented. The first, modularity maximization, was used to capture the communities which were internally dense and externally sparse (assortative) reflecting a segregated and autonomous organization.^71–73^ However, recently, it has been discussed that modularity maximization and related techniques may overlook some important and functionally relevant characteristics of neural circuits which exhibit non-assortative wiring.^74,75^ Because of this, we also used a weighted stochastic block model (WSBM), a generative modelling approach to describe a wider range of community structure topologies by explicitly considering patterned interactions between communities.^76,77^ In general, WSBM communities exhibit greater hemispheric symmetry, are spatially less compact than those derived from modularity maximization and more closely reflect functional networks.

Here, we applied both methods to capture the features of both assortative and non-assortative networks. For assortative modules, the modularity maximization algorithm as implemented in the brain connectivity toolbox (BCT; https://sites.google.com/site/bctnet)^68^ was used for each individual subject. At its core, the algorithm uses the Louvain method for community detection, which optimizes the modularity as the algorithm progresses.^73^ For computation, we used weighted and undirected connectivity matrices, resolution parameter greater than one and negative weights were treated symmetrically. Five thousand iterations were performed and the assignment of each region to a particular module was based on the maximum number of times/iterations a region was assigned to a module.^78^ Similarly, modules based on WSBM were computed using the script made available by Betzel *et al.*^77^ at http://tuvalu.santafe.edu/∼aaronc/wsbm/. WSBM is an extension which includes weighted edges in a stochastic block model, a probabilistic model of determining pairwise interactions between different nodes (see Aicher *et al.*^79,80^ and Faskowitz *et al.*^76^ for detailed mathematical description). The inputs of the algorithm consisted of the ‘*edge list*’ and the number of blocks (7), matching the number of modules obtained from the modularity algorithm. Again, 5000 iterations were performed for the assignment of each region to a particular module, as done for the maximum modularity computation.^78^

#### Controllability

The dynamics of complex systems depend upon the organization of the underlying network such that their elements can be associated with internal states that evolve over time.^81^ The control of a system dynamics is complex but relies heavily on the anatomical structure, or its topology.^82–84^ To estimate network-level control from structural connectivity, we calculated the average and modal controllability for the respective modules obtained from maximization modularity analysis. Both parameters were computed using the scripts available at https://complexsystemsupenn.com/codedata.^82,85^ Average controllability identifies a brain node or network that, on average, can steer the system into easily reachable and nearby states with little effort (i.e. minimal input energy). Modal controllability identifies a brain node or network that can drive the system into difficult-to-reach states (states that require substantial input energy). Here, we define a state to be the vector of neurophysiological activity at a single time point. From a cognitive perspective, these areas may be important in switching the brain between functions that require significant cognitive effort.^82^ For this study, as we were interested in observing the network-level control or the loss of it in AWS, we pooled the controllability values obtained from each node to obtain an average value for each module per group. We then compared each module from the AWS group to the same module from FS group (matched for age and sex to AWS) using the *t*-test for statistical significance. The controllability analysis was only carried out for the modules obtained from maximum modularity, as we were interested in observing if the variation in the (fewer) non-overlapping nodes between the groups might be the reason for loss of controllability in the AWS. For the modules obtained using the WSBM, the nodes in the modules for different groups were highly non-overlapping, making it ill-suited for the comparison between groups.

#### Network hubs

The weighted connectivity index between ROIs in the matrix was further analysed using algorithms implemented in the BCT.^86^ The network parameters of distance, clustering and centrality were computed to observe the influence over information transfer, principle hubs and network reorganization for segregation or integration. Here, network hubs were computed using two network measures—degree and betweenness centrality. A node is considered as a hub if its regional degree/betweenness centrality is substantially higher than the average network degree/betweenness.^87^ We computed hubs based on 1 and 2 standard deviations (SDs) from the average for each group. A concise explanation of these measures is presented below.

Degree of a node is the number of links it has with other nodes in the network. It is one of the fundamental network measures and is an obvious measure for computing hubs representing the importance of an individual node to network efficiency.^48^ The efficiency of a network here is a measure of how easily it exchanges information.^88^

Betweenness centrality of a node is the fraction of all shortest paths in the network that contains a given node and measures the extent to which the node lies on paths between other nodes. A node with higher betweenness centrality is considered a hub as it participates in a large number of shortest paths and has considerable influence in the network by virtue of its control over information passing between nodes.^89^

### Data availability statement

The raw, anonymized and defaced MRI along with the behavioural data could be made available upon reasonable request to the corresponding author and relevant Institutional Review Board approval needed for obtaining the patients’ data.

## Results

### Structural connectivity

**Figure 1:**
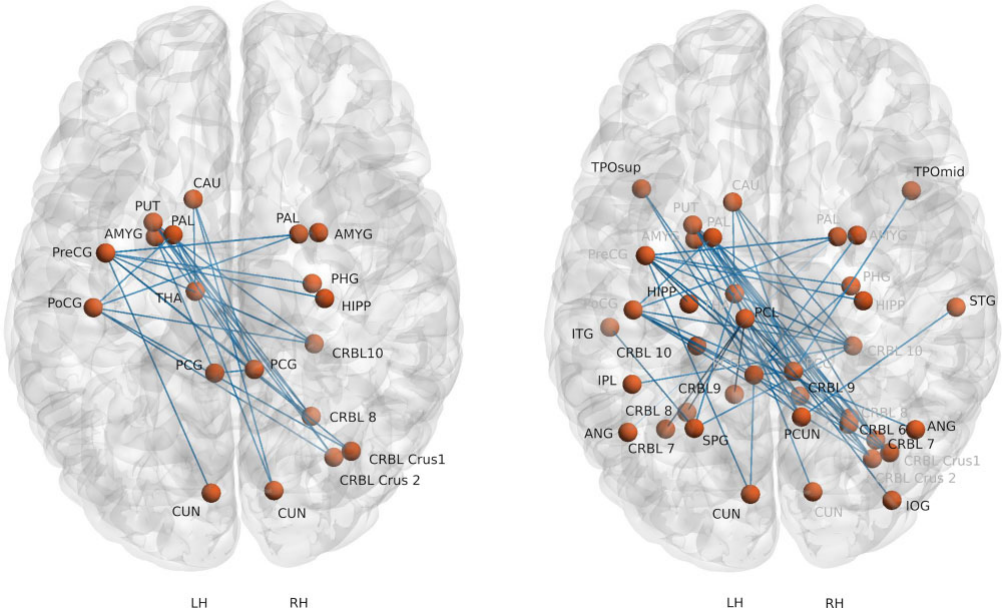
**Connectivity difference between adults who stutter and fluent speakers**. Reduced connectivity for the adults who stutter compared with fluent speakers depicted using the structural connectivity obtained using probabilistic tractography and compared using NBS. The figure on the left is thresholded at *P* = 0.020 and on the right is thresholded at *P* = 0.047. The background template is a Colin Brain with cerebellum (LH, left hemisphere; RH, right hemisphere) registered in MNI space.

From the NBS analysis, we observed a network encompassing cortical, subcortical and cerebellar (CRBL) regions which had significantly (*P* < 0.020) lower connectivity for AWS in comparison to FS (Fig. 1; Table 2); there were no instances of increased connectivity. Regions with reduced connectivity included pre-central gyrus (PreCG), postcentral gyrus (PoCG), posterior cingulate gyrus (PCG), cuneus (CUN), caudate (CAU), putamen (PUT), pallidum (PAL), amygdala (AMYG), hippocampus (HIPP), parahippocampal gyrus (PHG) and thalamus (THA); structures represented bilaterally included the AMYG, PAL, PCG and the CUN. All the CRBL regions were located on the right hemisphere and included Crus I, II and Lobules VIII, X. The cortical and subcortical regions were mostly distributed evenly with 9 on the left and 10 on the right side, however, of the 28 pairs showing reduced connectivity, only 4 were intra-hemispheric (in the left hemisphere) with the remaining 24 pairs comprising differences that were inter-hemispheric (see Table 2).

**Table 2:** Network-based statistics results detailing the networks having lower structural connectivity in AWS compared with FS

Left hemispheric network				Right hemispheric network			
Regions			*T*-stats values	Regions			*T*-stats values
Left	—	Left		Right	—	Right	
CAU	—	THA	**2.23**	PCG	—	IOG	*1.72*
PCG	—	AMYG	**2.05**		—	TPOmid	*1.79*
	—	CUN	*1.79*	PCUN	—	STG	*2.01*
	—	TPOsup	*1.99*				
PCL	—	CRBL7b	*2.09*				
	—	CRBL8	*1.94*				
	—	CRBL9	*2.46*				
PoCG	—	PAL	**2.24**				
	—	CRBL10	*1.87*				
PreCG	—	HIP	*1.81*				
	—	CUN	**2.22**				
SPG	—	THA	*1.77*				
	—	ITG	*1.88*				
**Inter-hemispheric network**							
**Regions**			***T*-stats values**	**Regions**			***T*-stats values**
**Left**	**—**	**Right**		**Left**	**—**	**Right**	
ANG	—	PAL	*1.74*	PreCG	—	HIP	**2.53**
CAU	—	CRBL7b	*1.89*		—	PHG	**2.46**
	—	PCG	**2.15**		—	AMYG	**2.61**
	—	CUN	**2.08**		—	PAL	**2.4**
	—	CRBL10	*1.97*		—	CRBLCrus1	**2.37**
IPL	—	PAL	*1.88*		—	CRBLCrus2	*1.87*
	—	CRBL10	*1.72*		—	CRBL8	**2.08**
PAL	—	CRBLCrus2	**2.91**		—	CRBL9	*1.74*
	—	PCG	**2.32**		—	CRBL10	**2.1**
	—	CUN	**2.49**	PUT	—	CRBLCrus1	*1.9*
	—	CRBL7b	*1.87*		—	CRBLCrus2	**2.87**
	—	CRBL8	**2.41**		—	PCUN	*1.74*
PCG	—	PCG	**2.19**		—	PCG	**2.54**
	—	PHG	*1.71*		—	CRBL7b	*1.84*
	—	AMYG	*1.87*		—	CRBL8	**2.2**
	—	PCUN	*2*		—	CRBL10	**2.3**
PCL	—	CRBLCrus2	*1.93*	PoCG	—	ANG	*1.87*
	—	CRBL8	*1.7*		—	PAL	**2.46**
	—	CRBL9	*1.86*		—	CRBLCrus1	**2.19**
	—	CRBL10	*1.95*		—	CRBLCrus2	**2.04**
SPG	—	CRBL10	*1.98*		—	CRBL6	*1.88*
TPOsup	—	PCUN	**2.24**		—	CRBL10	**2.27**
AMYG	—	PCG	**2.6**	THA	—	CRBL10	**2.33**

The *t*-stat values for significant differences at *P* < 0.020 are in bold font and for significant differences at *P* < 0.047 are in italic font.

A larger network with additional cortical, subcortical and CRBL regions was observed at a lower level of significance (*P* = 0.047) (Fig. 1, Table 2, *t*-values in bold). The additional reduced connectivity profiles were comprised of bilateral—angular gyrus (ANG), left—paracentral lobule (PCL), superior parietal gyrus (SPG), inferior parietal gyrus, inferior temporal gyrus (ITG), superior temporal gyrus (TPOsup) and right—inferior occipital gyrus (IOG), precuneus (PCUN) and the middle temporal gyrus (TPOmid). The additional subcortical and CRBL regions were the left—HIPP, CRBL Lobule VIIB, VIII, IX, X and right—CRBL VI, VIIB, IX.

### Bayesian power analysis

Using the Bayesian approach to test the group differences and effect size, we found that, out of all the NBS structural connectivity differences, the majority (73% in the lower threshold and 79% in the higher threshold) were of medium (>0.3) to large (>0.5) effect size. The details of the effect size distribution along with its modal value are presented as a Supplementary Figs. 1 and 2.

### Correlations with stuttering severity and self-rated anxiety

**Figure 2:**
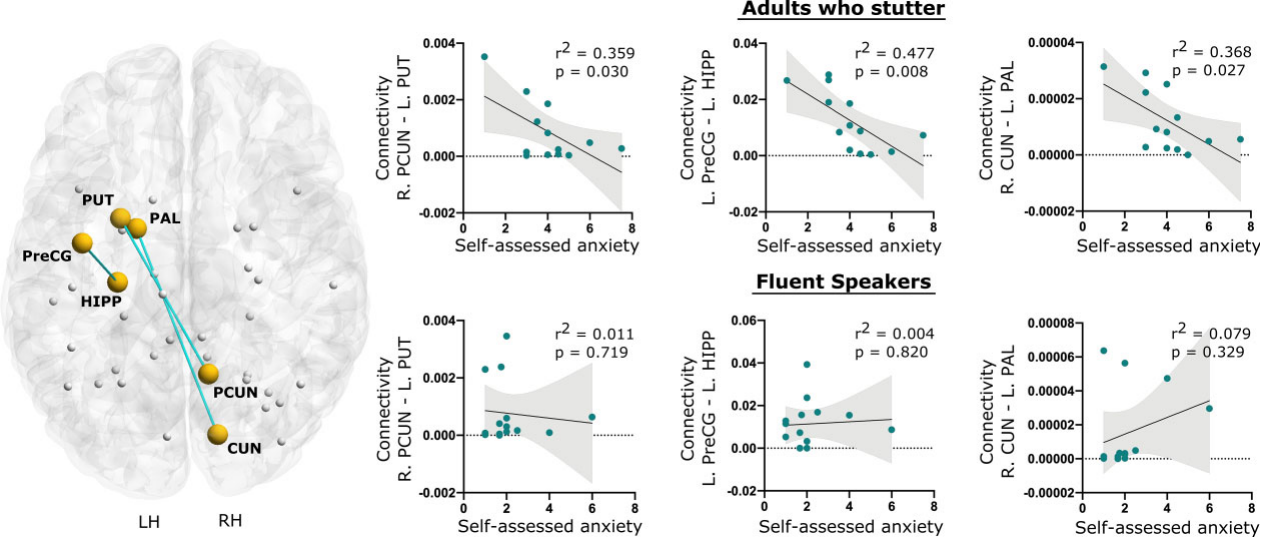
**Association to behavioural measures**. Connection between the regions in the network which were significantly negatively correlated with self-assessed anxiety in AWS and the corresponding correlations for the FS. *Y*-axis values represent the connectivity strength between the regions computed as the ratio of number of samples (or streamlines) that passes through those ROIs to all generated streamlines. The background template is a Colin Brain registered in MNI space.

We found a number of significant relationships between behaviour and connectivity for the self-rated anxiety scores of the AWS but not for FS (Fig. 2). Specifically, self-rated anxiety in AWS was negatively correlated with connectivity strength between right PCUN to left PUT, left PreCG to the left HIPP and right CUN to left PAL [AWS: *r*^2^ = 0.36 (*P* = 0.030), *r*^2^ = 0.48 (*P* = 0.008), *r*^2^ = 0.37 (*P* = 0.027); FS: *r*^2^ = 0.01 (*P* = 0.719), *r*^2^ = 0.004 (*P* = 0.820), *r*^2^ = 0.08 (*P* = 0.329), respectively]. However, both self-rated stuttering severity and severity obtained from the SSI-4 [highly correlated to each other (*r*^2^ = 0.61)] showed no relationship to the reduced connectivity in the sample.

### Network hubs

**Figure 3:**
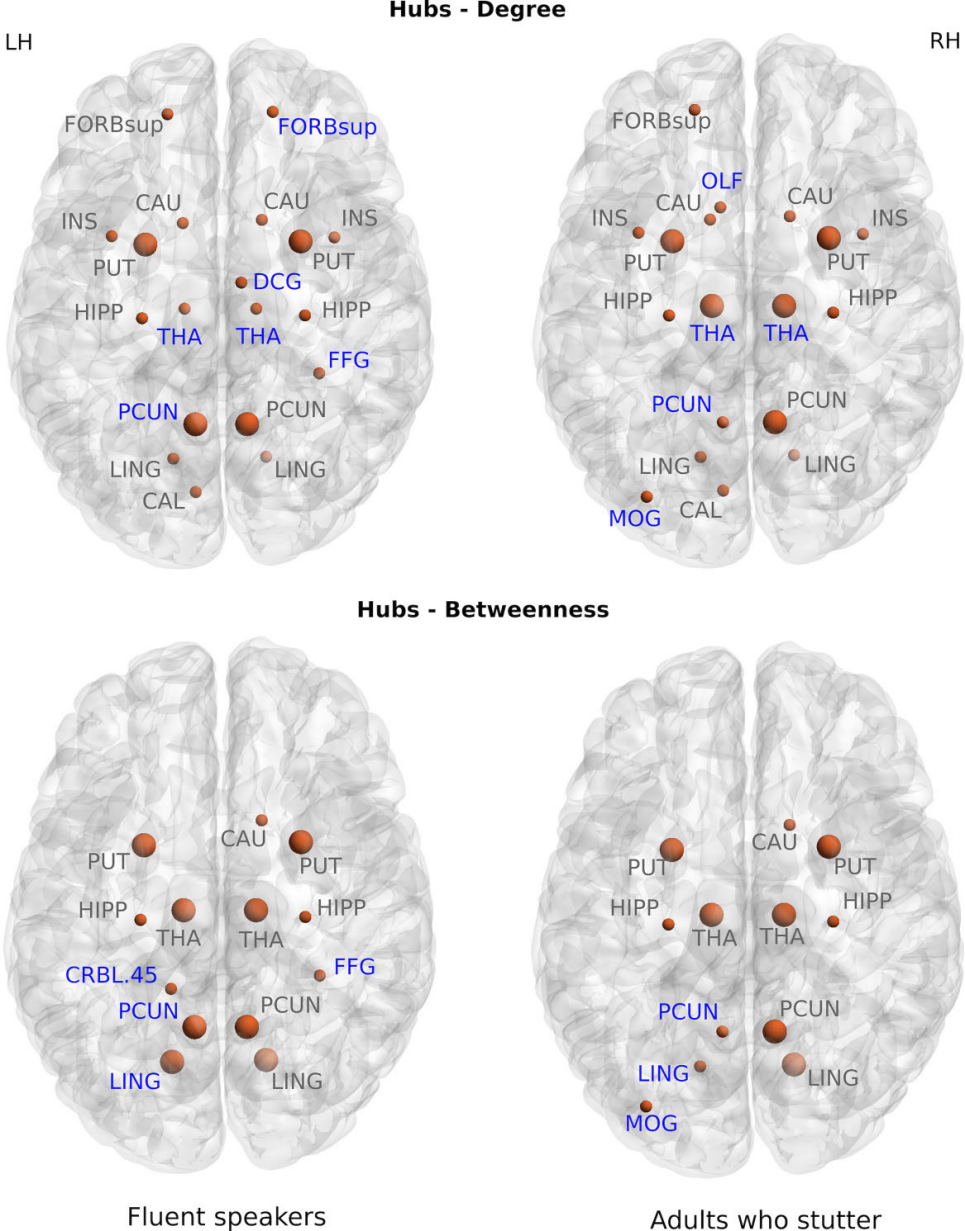
**Network hubs**. Network hubs, based on degree and betweenness centrality, for adults who stutter and fluent speakers based on 1 and 2 SDs from the average for each group, with blue highlights identifying differences between the groups. Larger nodes indicated hubs with more than 2 SDs from the average of the group.

From the graph theory analysis, significant differences in network hubs based on degree and betweenness centrality (*P* < 0.05, FDR corrected) were observed between the groups (Fig. 3). Four nodes for FS and five for AWS were identified as hubs whose nodal degrees were 2 SD above the average. For AWS, the bilateral THA nodes were more prominent with reduced strength for left PCUN node compared to the FS. At a less stringent threshold (1 SD) a number of other differences were noted in degree hubs including the right—superior orbital frontal gyrus (ORBsup), median cingulate and paracingulate gyri (DCG) and fusiform gyrus (FFG) for FS and left olfactory cortex and right middle occipital gyrus (MOG) for AWS. For hubs based on betweenness centrality, the left PCUN and lingual gyrus (LING) reflected reduced betweenness in AWS compared to FS. At the less stringent threshold, some hubs were missing for the AWS compared to the FS (CRBL 4 of 5 on the left, the FFG on the right) while the left MOG was present in AWS but not in the FS. Furthermore, in the group-wise comparison for each node, we found lower degree in left MOG and right THA and lower betweenness centrality in left LING, MOG, SPG and right PreCG and medial orbital frontal gyrus (ORBsupmed) in the AWS.

### Community structure—maximum modularity

**Figure 4:**
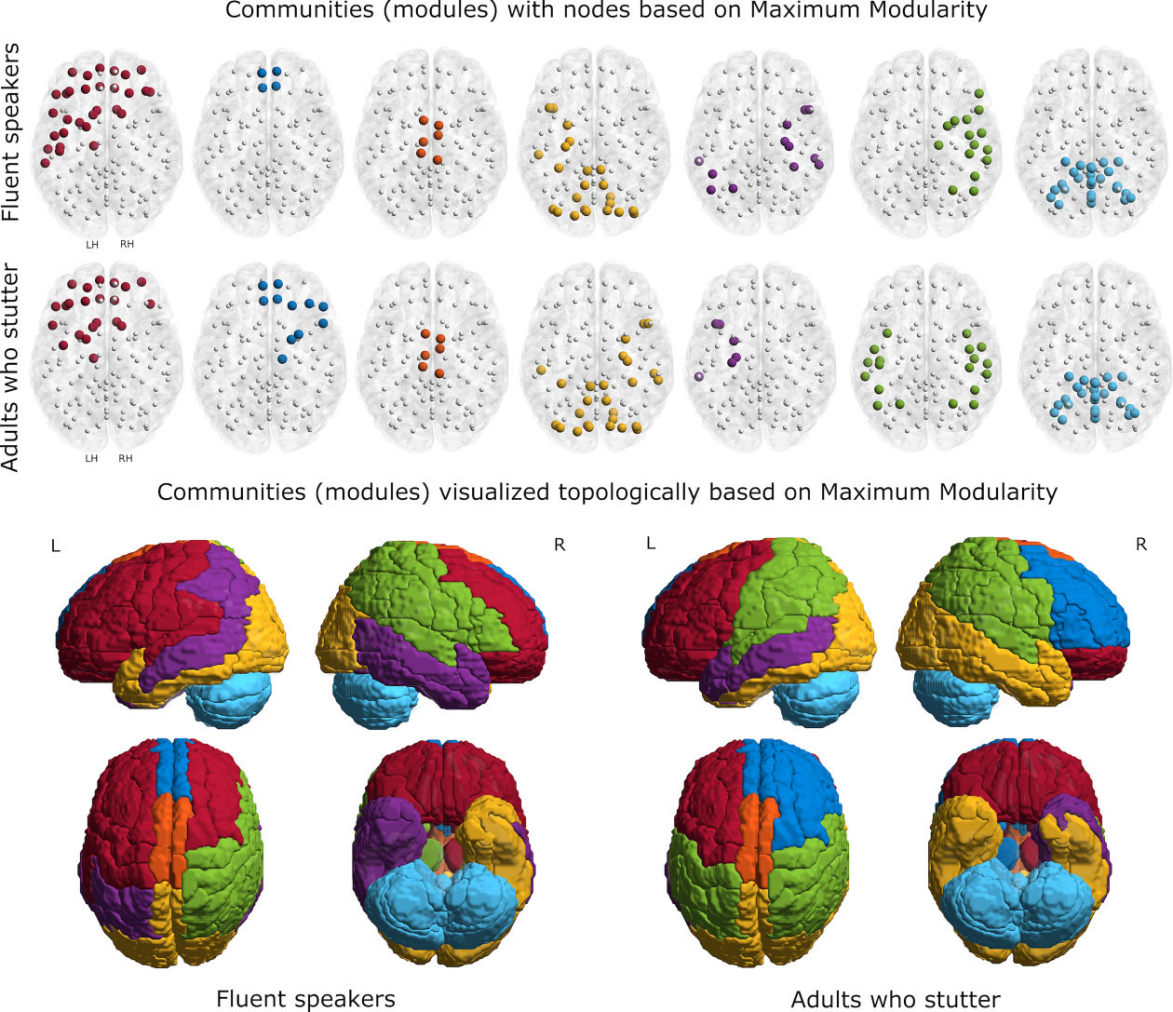
**Network modules—maximum modularity**. Network modules (shown in terms of nodes and topologically) of adults who stutter, and fluent speakers based on maximum modularity. Background template is a Colin Brain with cerebellum registered in MNI space. The details of the regions in each module are presented in Supplementary Table 1.

The optimization based on maximum modularity yielded seven non-overlapping communities (modules) for each of the groups. The modules and their associated regions are shown in Fig. 4 and detailed in Supplementary Table 1. Module (pseudo) names were assigned based on the cortical regions most prominently represented for the FS. Five of the seven modules were distinctly different between the groups. The two identical modules include the medial module encompassing bilateral supplementary motor area (SMA), the motor portion of the cingulum and the PCL (MEDIAL; in orange) and the CBM (in light blue). The modules that differed significantly for the groups included; (i) a Frontal module (FRONTAL; left > right, in red), (ii) an anterior medial module (A_M_F; in blue) extending into the right frontal area for the AWS, (iii) a posterior medio-temporal module (POST_MED_LAT; in gold) that included the right middle and inferior temporal area for AWS but not for the FS, (iv) a temporoparietal module (TEMP_PAR; in purple) with the bilateral organization for the FS but only left sided for the AWS and (v) a frontoparietal module (FRONTAL_PAR; in green) that was bilaterally represented in the AWS and only right-sided for the FS. Of particular note are the differences that include (i) the large FRONTAL module on the left hemisphere for FS which is split into two modules at the central sulcus for AWS, (ii) the fractionated dorsal auditory pathway on the left for AWS compared to FS, (iii) the bilateral parietal regions for AWS which is split across three modules for FS and (iv) the bilateral inferior temporal regions which is bilateral for AWS but is split into two modules for FS.

### Community structure—WSBM

**Figure 5:**
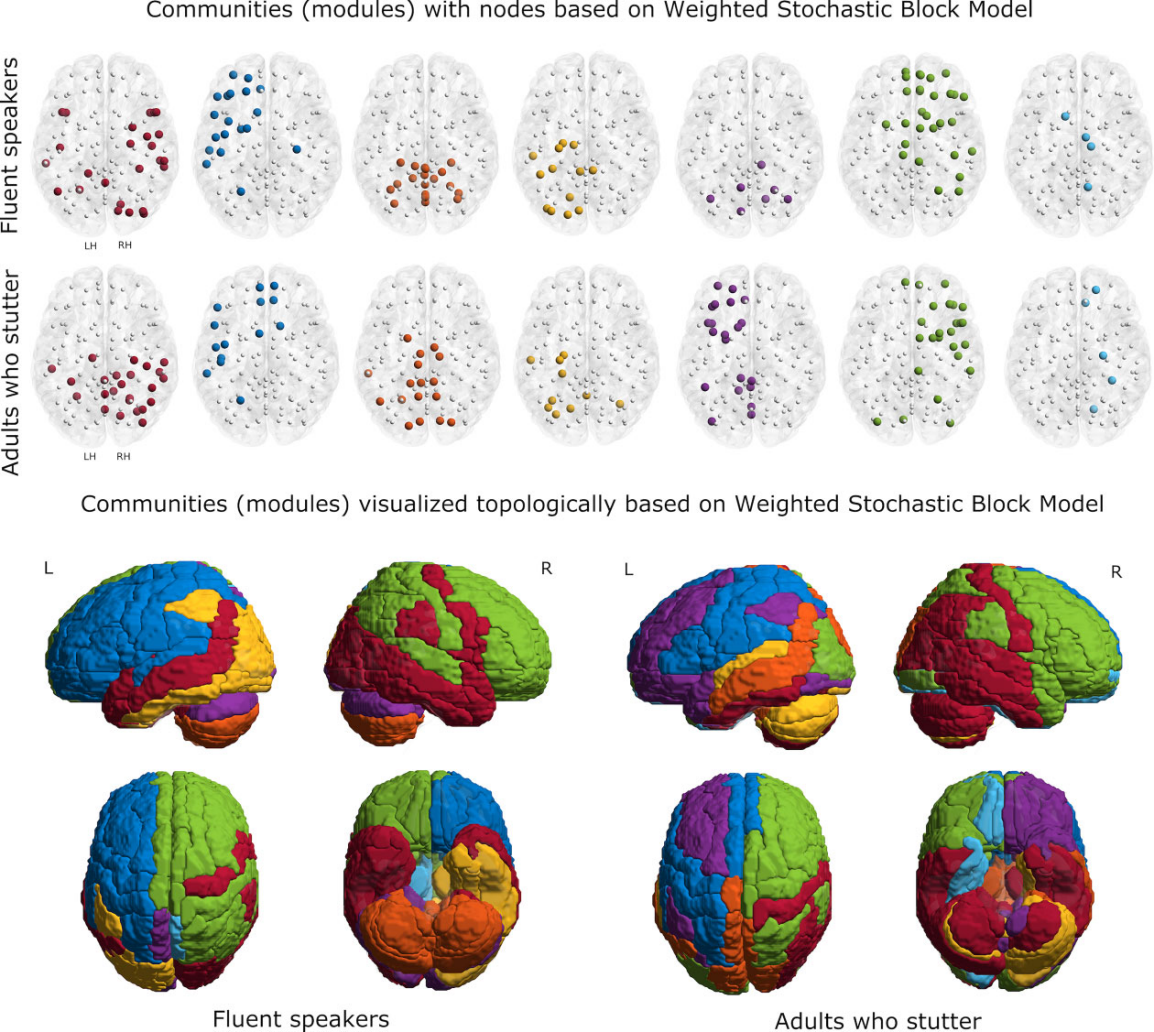
**Network modules—weighted stochastic block model**. Network modules (shown in terms of nodes and topologically) of adults who stutter and fluent speakers, based on weighted stochastic block modal. Background template is a Colin Brain with cerebellum registered in MNI space. The details of the regions in each module are presented in Supplementary Table 2.

Consistent with the more generative and less constrained approach, the WSBM resulted in more fractionated communities for both groups compared to the communities based on maximum modularity. The modules and their associated regions for FS and AWS are shown in Fig. 5 and detailed in Supplementary Table 2. Here, frontal and parietal regions (left hemisphere) were assigned to a single module for FS, but the same regions make up two modules for AWS, separating the pre-frontal cortex from frontal areas including the precentral and inferior frontal gyri. The right hemisphere frontoparietal region contains two modules for both groups. However, the sensorimotor portion of the modules (precentral and supramarginal gyri) was clustered differently. The CBM is more fractionated and predominantly lateralized for AWS but was organized bilaterally in a superior/inferior dimension for FS. In addition, the frontal and parietal midline medial structures clustered with the right frontoparietal module for the FS but were more fractionated for the AWS with the left and right sides clustering to different modules.

### Controllability

For controllability, significant differences were noted primarily in the frontal module. The AWS showed higher average controllability (AWS: 1.05126, FS: 1.04360, *P* = 0.0129) and lower modal controllability (AWS: 0.95719, FS: 0.96233, *P* = 0.0222) compared with the FS in this module. There were no significant differences for the remaining modules (see Table 3 for details).

**Table 3:** Controllability measures for both groups for each module

Measures	Modules	AWS (mean ± SD)	FS (mean ± SD)	*P*-value (* < 0.05)
Average controllability	FRONTAL	1.05126 ± 0.011	1.04360 ± 0.010	0.0129*
	A_M_F	1.04440 ± 0.012	1.03560 ± 0.003	0.1722
	MEDIAL	1.03530 ± 0.007	1.03222 ± 0.005	0.3977
	POST_MED-LAT	1.04220 ± 0.012	1.04397 ± 0.012	0.6038
	TEMP_PAR	1.04286 ± 0.009	1.03628 ± 0.007	0.1093
	FRONTAL_PAR	1.03479 ± 0.006	1.03847 ± 0.010	0.1968
	CBM	1.05654 ± 0.018	1.05492 ± 0.014	0.7178
Modal controllability	FRONTAL	0.95719 ± 0.007	0.96233 ± 0.008	0.0222*
	A_M_F	0.96123 ± 0.009	0.96828 ± 0.003	0.1754
	MEDIAL	0.96934 ± 0.006	0.97137 ± 0.005	0.5156
	POST_MED-LAT	0.96348 ± 0.009	0.96281 ± 0.009	0.7966
	TEMP_PAR	0.96228 ± 0.007	0.96769 ± 0.006	0.1007
	FRONTAL_PAR	0.96870 ± 0.005	0.96579 ± 0.009	0.2345
	CBM	0.95375 ± 0.014	0.95458 ± 0.011	0.807

AWS, adults who stutter; FS, fluent speakers; SD, standard deviation; A_M_F, anterior medial module; POST_MED-LAT, posterior medio-temporal module; TEMP_PAR, temporoparietal module; FRONTAL_PAR, frontoparietal module; CBM, cerebellum module.

* *P-*values < 0.05 for statistical significance.

## Discussion

In the current study, we assessed the structural connectome in a small cohort of AWS using network properties derived from DWI. Consistent with the literature and extended clinical features associated with the disorder, we found wide-spread reduced connectivity in both motor and non-motor brain areas. Reduced connectivity, predominantly inter-hemispheric, was observed in limbic areas, in regions associated with the default mode network (DMN) and in non-motor areas of the CBM. Graph theoretical analysis identified differences in network hubs suggesting reduced subnetwork efficiency as well as changes in community structure that are consistent with network-level adaptations in AWS. An exploratory analysis of network controllability found preliminary evidence of frontal lobe changes related to speech motor control and executive function. In order to offset the lack of power due to the small sample size, we subjected the data from the two groups to a Bayesian analysis to examine effect sizes for the connectivity results. Interestingly, we found a range of effect sizes that appear to reflect the significant clinical heterogeneity associated with the disorder.

### Structural connectivity

For stuttering, a persistent neurodevelopmental disorder with multiple aetiologies, risk factors and socioemotional and cognitive features, we expected a wide range of structural changes. Using NBS (Fig. 1), we observed wide-spread reduced structural connectivity differences in AWS, indicating spatially distributed degradation in white matter connections compared to FS; increased connectivity was not observed. Reduced connectivity from the left pre-central region was predominantly interhemispheric to the right globus pallidus (GP), HIPP, parahippocampus, AMYG and CBM (Crus I, II and Lobules VIII, X). The left pre-central region is often under-activated in functional studies when individuals who stutter produce fluent speech.^6–8,11,90,91^ Interestingly, with the exception of the GP and Lobule VIII of the CBM, reduced connection strength was associated with brain regions associated with cognitive, memory and emotional processing rather than solely related to sensorimotor control.^92,93^ The connectivity of the PoCG on the left with the right GP and CBM (Lobule X and Crus I, II) was reduced as well. Reduced connectivity of left PoCG to right CBM (Lobule X) and bilateral GP has been associated with auditory and somatosensory feedback in fMRI studies of non-stuttering adults.^94,95^ The reduced connection between GP (which has been associated with motor learning^96^) and left posterior central gyrus may be a manifestation of the often reported deficiency in sensorimotor integration which impacts sensorimotor learning in AWS.^97–99^

However, the other brain regions associated with the reduced connectivity with the left pre- and post-central gyrus (AMYG, HIPP and parahippocampus) have not been widely reported. In this regard, many of the areas of reduced structural connectivity are components of the DMN, including the posterior cingulate cortex and PCUN, medial pre-frontal cortex, ANG, temporoparietal junction, anterior and lateral temporal cortex, HIPP, parahippocampus, posterior inferior parietal lobe and the retrosplenial cortex, medial pre-frontal cortex from the frontal pole to the anterior cingulate.^100–104^ A major area of reduced connectivity was found between the right PCG and left—AMYG, CAU, GP and PUT. In addition to being a hub of the DMN the right PCG has been associated with more cognitive-related conditions for speech such as semantic processing and picture description.^105,106^ The contribution of left AMYG to the bilateral PCG is of interest in its potential role in emotional regulation, specifically associated with threat detection^107^ and response to emotional valence.^108^ Resting state hypoconnectivity between the left AMYG and PCG has been reported in older children with anxiety disorder.^109^ All these regions are implicated in cognitive, emotional and memory-related processes^110^ and goal-oriented tasks involving interpersonal experiences and interaction.^104^ Even at the more lenient threshold no right hemisphere motor regions (pre-central, pre-motor), no left inferior frontal or superior/medial temporal regions (only the right TPOsup) showed reduced structural connectivity, regions that are routinely reported in functional neuroimaging studies of individuals who stutter when producing fluent speech.^111,112^ The current results suggest that reduced structural connectivity is manifest in multiple brain regions potentially impacting a wide range of behaviour.

### Network hubs

Analysis of network hubs provide insight into neural integration and communication that impacts all behavioural domains. In the current study, we focussed on nodal degree, as a measure of the number of connections at a node, and betweeness centrality, as a measure of the influence of a structural node over the flow of neural information. The network hubs (Fig. 3) obtained using nodal degree and betweenness centrality differed for the two groups. The left PCUN was reduced for both hub measures for the AWS while the bilateral THA was more prominent for nodal degree compared to the FS. The reduced degree and betweenness suggests that the left PCUN has less influence over the flow of information associated with its functional role for the AWS compared to the FS. As a specialized hub of the DMN,^104,113^ the PCUN provides a bridge between other nodes and plays a crucial role in engaging functional integration across multiple cognitive-related networks^114,115^ engaged for autobiographical memory retrieval,^116^ reward outcome monitoring,^117^ emotional stimulus processing^118^ and has increased functional connectivity with the left frontoparietal network.^113^ Examining resting state connectivity differences in children and AWS, the DMN has been shown to exhibit decreased functional connectivity with pre-frontal areas.^119^ While the AWS were missing only the left PCUN, it can be assumed that this missing node puts a substantial strain on information transfer in much of the left hemispheric network nodes. In contrast, the bilateral THA was more heavily represented for nodal degree for the AWS, suggesting a more prominent role for the THA and a potential imbalance in the weighting of sensory input in a wide range of behaviours. Considering the hubs results together, it is possible that loss of left PCUN creates nodal overload in the THA, impacting information flow through the network. And, in turn, this overload may have a substantial effect in the information transfer to cortical regions related to both the THA and the DMN. Overall, the network hub results suggest that there are differences in the structural organization that underlies multisystem neural communication for the AWS compared to the FS.

### Community structures

We also compared network organization of topologically related modules to evaluate differences in community structure for the groups resulting from the white matter changes.^68,120^ Community structure was evaluated with two different approaches, maximum modularity (Fig. 4) and WSBM (Fig. 5), providing complementary and unique information on network architecture. Maximum modularity identified seven modules that reflect densely connected brain regions that are sparsely connected to other communities of brain regions. Of the seven modules identified using maximum modularity for AWS, two contained the same regions as those derived for FS. The similar modules encompassed the entire CBM and midline non-primary motor and sensory areas (bilateral supplementary motor and cingulate motor areas, and the PCL). For the CBM, despite reduced connectivity with multiple cortical and subcortical regions, there were no differences in the modular organization for both groups. The CBM has been implicated in increased functional activation in AWS^8,121^ with changes in CRBL connectivity^122^ and along with cortical structures possibly underlying recovery^123,124^ or compensation related to reduced stuttering severity.^6,21,125^ The lack of difference between the groups suggests that structurally the CBM is relatively unaffected by persistent stuttering; differences are apparently more associated with tracts running to and from rather than a change in its intrinsic organization^52^. For the non-primary motor regions, a portion of the frontal aslant tract runs from supplementary motor area (SMA) to inferior frontal gyrus and has been implicated in stuttering.^126,127^ However, no differences with either the SMA or cingulate motor areas (CMAs) for the AWS was observed, again suggesting a problem with connectivity rather than intrinsic organization of the respective sensorimotor areas.

The remaining modules differed in multiple ways. The large left hemisphere frontal module for the FS contained areas related to speech motor control including those associated with auditory and somatosensory feedback [Rolandic operculum (OR), insula, supramarginal gyrus, PoCG and TPOsup]. In contrast, these same regions for the AWS were part of a large and bilateral parietal module. In contrast to the FS, the module containing the anterior cingulum and superior medial frontal gyrus, areas associated with numerous cognitive functions, including decision making and memory with extensive connections to limbic regions, was associated with additional right hemisphere pre-frontal and subcortical regions for the AWS. There were also multiple examples of a differential lateralization in the modules involving limbic structures (HIPP, AMYG and PHG). Overall, the modular organization of 5 of the 7 modules suggests a differential and presumably compensatory structural organization for the AWS compared to the FS.

Community structure from the WSBM analysis provides a more complex and interesting picture. WSBM is a generative modelling approach, which not only serves as a description of the network structure but also encompasses wide range of other topological features by explicitly considering patterned interactions between communities.^76^ Overall, WSBM resulted in more fractionated communities in AWS, especially in the left hemisphere, compared to FS. The frontal–parietal–superior temporal regions subserve a range of speech, language and cognitive functions and the internal organization for the FS suggests processing within a large and distributed module. In contrast, for the AWS, the same brain regions on the left hemisphere are made up of three different modules. The fractionated organization for AWS suggests more localized processing in the individual pre-frontal, frontoparietal and superior temporal modules, potentially resulting in the processing of information that is more heavily dependent on communications across modules. A similar fractionation was found for the anterior and mid portion of the medial cortex. For the FS, the medial portion, which includes much of the DMN, is part of a large right hemisphere module that encompasses the frontal and parietal cortex. For the AWS, the same structures are part of the left frontoparietal region, the middle temporal region and the right frontoparietal region. While functional interpretations are difficult from structural data, the fractionation suggests that the processing of information for the AWS engages more short-distance connections, which are more energetically costly and inefficient compared to long-distance connections because they increase the number of processing steps.^128,129^ In contrast to the results from maximum modularity, the CRBL organization differs considerably for the groups. For the most part, the CRBL regions within a module are bilateral for the FS, while for the AWS they are not, suggesting a decoupling of CRBL organization. In addition, CRBL regions for the AWS are more extensively distributed to cortical, subcortical and limbic areas than for the FS. Finally, the medial motor area, DMN and limbic structures all clustered differently in the two groups, consistent with an overall change in network organization to modulate or modify emotional and transmodal processing.

### Controllability

The controllability results were confined to a single module that included much of the frontal cortex. For the AWS, the average controllability for this module was higher and the modal controllability was lower than for the FS. Recall that *average controllability* identifies brain areas that, on average, can steer the system into different states with little effort (that is, little input energy) and modal controllability identifies a brain node or network that can drive the system into difficult-to-reach states (states that a require substantial input energy). In this regard, it is interesting to consider the brain areas that appear to drive the controllability differences. For the AWS—the RO, Insula, SupraMarginal gyrus, Heschl's gyrus, superior temporal gyrus (STG) were missing from the left hemisphere along with the superior and middle frontal regions in the right hemisphere. The left hemisphere regions are specifically related to sensorimotor functions, including speech motor production and sensory feedback, while the right hemisphere regions are associated with cognitive and socioemotional functions, including social anxiety.^130–134^ The decoupling of these brain areas may reflect a functional adaptation facilitating the production of fluent speech in AWS, albeit in an alternative manner from FS. Consistent with this interpretation is the documented differences in multiple aspects of the fluent speech of individuals who stutter.^53,135–138^ That is, even when AWS produce fluent speech, the kinematic characteristics and sensorimotor mechanisms used to produce and control that speech have been shown to differ, and this difference is often explained in terms of a variable or unstable control system.^139–144^ In contrast, modal controllability was lower for AWS, suggesting a constraint on the ability to deal with demanding tasks, especially those associated with cognitive effort.^82^ Interestingly, the right hemisphere frontal regions for the AWS are part of the medial pre-frontal module, which supports a range of higher-level functions related to social cognition and social interactions.^145^ The greater association of AWS with social anxiety disorder^26,27,31^ may be a manifestation of the lower modal controllability in the pre-frontal cortex.

### Statistical power and clinical heterogeneity

Stuttering is a developmental disorder with genetic and epigenetic (environmental) factors impacting brain and behavioural development. These varied influences on the disorder lead to one of its typical features, phenotypic heterogeneity.^146–149^ While AWS display some form of speech dysfluency, less common are associated problems with language, executive and socioemotional function. Our purpose in the current study was to examine whole-brain connectivity for evidence that was more consistent with the clinical heterogeneity of the disorder rather than focus primarily on the sensorimotor/speech motor aspects. Our results clearly illustrate connectivity changes that are associated with both the sensorimotor character of the disorder as well as socioemotional and cognitive concomitants. Interestingly, our use of Bayesian statistics to evaluate the effect sizes of the structural connectivity differences appear to reflect the phenotypic heterogeneity that characterizes the disorder. That is, the larger effect sizes, associated with more robust differences, were found for areas directly involved in the speech motor process. The more moderate effects were associated with brain areas that, while impacting speech production, are not directly related to the sensorimotor action; areas such as the AMYG, HIPP, parahippocampus, CUN, PCUN and posterior cingulate. Overall, a Bayesian procedure to assess effect size improved the ability to interpret the results from our small sample and provided a more complete picture of the individual differences in the consequences of the neurodevelopmental disorder.

## Conclusion

The current whole-brain analysis identified reduced connectivity for AWS in brain areas that extend beyond the speech motor regions. Areas such as the PCUN, HIPP and CUN are associated with processes involving behavioural inhibition, forming relational memories, episodic memories, affective responses and working memory. Recent work suggests that there is an association between memory control deficits and affect,^150^ and that reduced connectivity in these regions may be related to socioemotional processing. Also consistent with this idea, we showed reductions in connectivity that are correlated with subjective ratings of anxiety. Non-speech regions had reduced connectivity to left hemisphere sensorimotor areas, indicating that they may eventually influence the speech motor process itself. Finally, controllability differences in the functional utility of the frontal cortex in switching brain states, potentially related to fluent speech production and cognitive and emotional processing. While there are no data directly addressing whether the reduced connectivity is a cause or an effect of chronic stuttering, resting state connectivity differences exist in young children who stutter in major brain networks for situational, emotional and attentional processing^119^ suggesting that reductions in structural connectivity between these regions and the speech motor system may be an early biomarker of the disorder. Wide-spread network changes seen in this study reflect the consequences of a chronic neurodevelopmental disorder and its social repercussions.

## Supplementary Material

fcac058_Supplementary_Data

## Abbreviations

AWS =: adults who stutter
BCT =: brain connectivity toolbox
DMN =: default mode network
DWI =: diffusion-weighted images
FS =: fluent speakers
FSL =: FMRIB software library
MNI =: Montreal Neurosciences Institute
MPRAGE =: Magnetization-prepared rapid acquisition with gradient echo
NBS =: network-based statistic
ROI =: region of interest
SSI-4 =: Stuttering Severity Instrument, fourth edition
TE =: echo time
TR =: repetition time
WSBM =: weighted stochastic block model
