# Supplementary material for: Structural brain network topological alterations in stuttering adults
Source: Brain Commun. 2022 Mar 10;4(2):fcac058. doi: 10.1093/braincomms/fcac058 (PMC8971894; doi:10.1093/braincomms/fcac058)
Supplement: fcac058_Supplementary_Data [file fcac058_supplementary_data.pdf]

Structural brain network topological alterations in stuttering adults

Vincent L. Gracco[1,2], Anastasia G. Sares[3], Nabin Koirala[1*]

[1]Haskins Laboratories, New Haven, CT, USA

[2] School of Communication Sciences & Disorders, McGill University, Montreal, Canada

[3]Department of Psychology, Concordia University, Montreal, Canada

Supplementary Materials

Fluent Speakers	Adults who stutter	Fluent Speakers continue	Adults who stutter continue
**FRONTAL**	**FRONTAL**	Cuneus_R	Lingual_R
Precentral_L	Precentral_L	Lingual_L	Occipital_Sup_L
Frontal_Sup_L	Frontal_Sup_L	Lingual_R	Occipital_Sup_R
Frontal_Sup_R	Frontal_Sup_Orb_L	Occipital_Sup_L	Occipital_Mid_L
Frontal_Sup_Orb_L	Frontal_Sup_Orb_R	Occipital_Sup_R	Occipital_Mid_R
Frontal_Sup_Orb_R	Frontal_Mid_L	Occipital_Mid_L	Occipital_Inf_L
Frontal_Mid_L	Frontal_Mid_Orb_L	Occipital_Mid_R	Occipital_Inf_R
Frontal_Mid_R	Frontal_Mid_Orb_R	Occipital_Inf_L	Fusiform_L
Frontal_Mid_Orb_L	Frontal_Inf_Oper_L	Occipital_Inf_R	Fusiform_R
Frontal_Mid_Orb_R	Frontal_Inf_Tri_L	Fusiform_L	Precuneus_L
Frontal_Inf_Oper_L	Frontal_Inf_Orb_L	Precuneus_L	Precuneus_R
Frontal_Inf_Tri_L	Frontal_Inf_Orb_R	Precuneus_R	Temporal_Pole_Sup_R
Frontal_Inf_Orb_L	Olfactory_L	Temporal_Pole_Sup_L	Temporal_Mid_R
Frontal_Inf_Orb_R	Olfactory_R	Temporal_Pole_Mid_L	Temporal_Pole_Mid_R
Rolandic_Oper_L	Frontal_Mid_Orb_L	Temporal_Inf_L	Temporal_Inf_L
Olfactory_L	Frontal_Mid_Orb_R	**FRONTAL_PAR**	Temporal_Inf_R
Olfactory_R	Rectus_L	Precentral_R	**FRONTAL_PAR**
Frontal_Mid_Orb_L	Rectus_R	Frontal_Inf_Oper_R	Precentral_R
Frontal_Mid_Orb_R	Caudate_L	Frontal_Inf_Tri_R	Rolandic_Oper_L
Rectus_L	Caudate_R	Rolandic_Oper_R	Rolandic_Oper_R
Rectus_R	Putamen_L	Insula_R	Insula_L
Insula_L	Pallidum_L	Postcentral_R	Insula_R
Postcentral_L	Thalamus_L	Parietal_Sup_R	Postcentral_L
SupraMarginal_L	**A_M_F**	Parietal_Inf_R	Postcentral_R
Caudate_L	Frontal_Sup_R	SupraMarginal_R	Parietal_Sup_L
Caudate_R	Frontal_Mid_R	Angular_R	Parietal_Sup_R
Putamen_L	Frontal_Inf_Oper_R	Putamen_R	Parietal_Inf_L
Pallidum_L	Frontal_Inf_Tri_R	Pallidum_R	Parietal_Inf_R
Thalamus_L	Frontal_Sup_Medial_L	Thalamus_R	SupraMarginal_L
Heschl_L	Frontal_Sup_Medial_R	Heschl_R	SupraMarginal_R
Temporal_Sup_L	Cingulum_Ant_L	Temporal_Sup_R	Angular_L
**A_M_F**	Cingulum_Ant_R	**CBM**	Angular_R
Frontal_Sup_Medial_L	Putamen_R	Cerebelum_Crus1_L	Heschl_L

Frontal_Sup_Medial_R	Pallidum_R	Cerebelum_Crus1_R	Heschl_R
Cingulum_Ant_L	Thalamus_R	Cerebelum_Crus2_L	Temporal_Sup_L
Cingulum_Ant_R	**MEDIAL**	Cerebelum_Crus2_R	Temporal_Sup_R
**MEDIAL**	Supp_Motor_Area_L	Cerebelum_3_L	**CBM**
Supp_Motor_Area_L	Supp_Motor_Area_R	Cerebelum_3_R	Cerebelum_Crus1_L
Supp_Motor_Area_R	Cingulum_Mid_L	Cerebelum_4_5_L	Cerebelum_Crus1_R
Cingulum_Mid_L	Cingulum_Mid_R	Cerebelum_4_5_R	Cerebelum_Crus2_L
Cingulum_Mid_R	Paracentral_Lobule_L	Cerebelum_6_L	Cerebelum_Crus2_R
Paracentral_Lobule_L	Paracentral_Lobule_R	Cerebelum_6_R	Cerebelum_3_L
Paracentral_Lobule_R	**POST_MED_LAT**	Cerebelum_7b_L	Cerebelum_3_R
**POST_MED_LAT**	Cingulum_Post_L	Cerebelum_7b_R	Cerebelum_4_5_L
Cingulum_Post_L	Cingulum_Post_R	Cerebelum_8_L	Cerebelum_4_5_R
Cingulum_Post_R	Hippocampus_R	Cerebelum_8_R	Cerebelum_6_L
Hippocampus_L	ParaHippocampal_R	Cerebelum_9_L	Cerebelum_6_R
ParaHippocampal_L	Amygdala_R	Cerebelum_9_R	Cerebelum_7b_L
Amygdala_L	Calcarine_L	Cerebelum_10_L	Cerebelum_7b_R
Calcarine_L	Calcarine_R	Cerebelum_10_R	Cerebelum_8_L
Calcarine_R	Cuneus_L	Vermis_1_2	Cerebelum_8_R
Cuneus_L	Cuneus_R	Vermis_3	Cerebelum_9_L
**TEMP_PAR**	**TEMP_PAR**	Vermis_4_5	Cerebelum_9_R
Hippocampus_R	Hippocampus_L	Vermis_6	Cerebelum_10_L
ParaHippocampal_R	ParaHippocampal_L	Vermis_7	Cerebelum_10_R
Amygdala_R	Amygdala_L	Vermis_8	Vermis_1_2
Fusiform_R	Temporal_Pole_Sup_L	Vermis_9	Vermis_3
Parietal_Sup_L	Temporal_Mid_L	Vermis_10	Vermis_4_5
Parietal_Inf_L	Temporal_Pole_Mid_L		Vermis_6
Angular_L			Vermis_7
Temporal_Pole_Sup_R			Vermis_8
Temporal_Mid_L			Vermis_9
Temporal_Mid_R			Vermis_10
Temporal_Pole_Mid_R			
Temporal_Inf_R			

Supplementary Table 1: Regions associated with each module obtained using Maximum modularity algorithm as depicted in figure 4. Here L and R indicates Left and Right hemispheres and color codes are corresponding to module colors in the figure.

Fluent Speakers	Adults who stutter	Fluent Speakers continue	Adults who stutter continue
**F_T_P**	**P_T_CBM**	Vermis_9	Cerebelum_Crus1_L
Precentral_R	Hippocampus_R	Vermis_10	Cerebelum_Crus2_L
Frontal_Inf_Oper_R	Calcarine_R	**L_CIG**	Cerebelum_7b_L
Rolandic_Oper_R	Occipital_Mid_R	Cingulum_Post_L	Cerebelum_7b_R
ParaHippocampal_R	Fusiform_L	Cingulum_Post_R	Cerebelum_10_L
Amygdala_R	Postcentral_R	Hippocampus_L	Vermis_9
Calcarine_R	Parietal_Inf_R	ParaHippocampal_L	**L_FTP**
Cuneus_R	Angular_R	Cuneus_L	Frontal_Sup_L
Occipital_Sup_R	Thalamus_L	Lingual_L	Frontal_Sup_Orb_L
Occipital_Mid_R	Temporal_Sup_R	Occipital_Sup_L	Frontal_Mid_L
Occipital_Inf_R	Temporal_Mid_R	Occipital_Mid_L	Frontal_Mid_Orb_L
Fusiform_R	Temporal_Inf_L	Occipital_Inf_L	Frontal_Inf_Orb_L
SupraMarginal_R	Temporal_Inf_R	Fusiform_L	Olfactory_L
Angular_L	Cerebelum_Crus1_R	Parietal_Inf_L	Rectus_L
Heschl_L	Cerebelum_Crus2_R	Thalamus_L	Insula_L
Heschl_R	Cerebelum_3_R	Temporal_Inf_L	Amygdala_L
Temporal_Pole_Sup_L	Cerebelum_4_5_R	**S_CBM**	Occipital_Inf_L
Temporal_Pole_Sup_R	Cerebelum_6_R	Calcarine_L	Parietal_Inf_L
Temporal_Mid_L	Cerebelum_8_L	Lingual_R	Caudate_L
Temporal_Mid_R	Cerebelum_8_R	Precuneus_L	Putamen_L
Temporal_Pole_Mid_L	Cerebelum_9_R	Cerebelum_Crus1_L	Temporal_Pole_Sup_L
Temporal_Pole_Mid_R	Cerebelum_10_R	Cerebelum_Crus1_R	Temporal_Pole_Mid_L
Temporal_Inf_R	Vermis_1_2	Cerebelum_3_R	Cerebelum_3_L
Cerebelum_4_5_L	Vermis_4_5	Cerebelum_6_R	Cerebelum_9_L
Cerebelum_6_L	Vermis_6	**R_FTP**	Vermis_3
Vermis_4_5	**L_FTP**	Frontal_Sup_R	Vermis_7
**L_FTP**	Precentral_L	Frontal_Sup_Orb_R	Vermis_8
Precentral_L	Frontal_Inf_Oper_L	Frontal_Mid_R	Vermis_10
Frontal_Sup_L	Frontal_Inf_Tri_L	Frontal_Mid_Orb_R	**R_FTP**
Frontal_Sup_Orb_L	Rolandic_Oper_L	Frontal_Inf_Tri_R	Precentral_R
Frontal_Mid_L	Supp_Motor_Area_L	Frontal_Inf_Orb_R	Frontal_Sup_R
Frontal_Mid_Orb_L	Frontal_Sup_Medial_L	Supp_Motor_Area_L	Frontal_Mid_R
Frontal_Inf_Oper_L	Frontal_Sup_Medial_R	Supp_Motor_Area_R	Frontal_Mid_Orb_R
Frontal_Inf_Tri_L	Cingulum_Ant_L	Olfactory_R	Frontal_Inf_Oper_R
Frontal_Inf_Orb_L	Cingulum_Ant_R	Frontal_Sup_Medial_L	Frontal_Inf_Tri_R
Rolandic_Oper_L	Postcentral_L	Frontal_Sup_Medial_R	Frontal_Inf_Orb_R
Olfactory_L	Parietal_Sup_L	Frontal_Mid_Orb_L	Rolandic_Oper_R
Rectus_L	SupraMarginal_L	Frontal_Mid_Orb_R	Supp_Motor_Area_R
Insula_L	Caudate_R	Rectus_R	Olfactory_R
Hippocampus_R	Heschl_L	Insula_R	Frontal_Mid_Orb_L
Amygdala_L	**P_MED_TEMP**	Cingulum_Ant_L	Frontal_Mid_Orb_R
Postcentral_L	Cingulum_Mid_L	Cingulum_Ant_R	Insula_R
Parietal_Sup_L	Cingulum_Mid_R	Cingulum_Mid_L	Amygdala_R
SupraMarginal_L	Cingulum_Post_L	Postcentral_R	Calcarine_L
Pallidum_L	Cingulum_Post_R	Parietal_Sup_R	Occipital_Mid_L
Temporal_Sup_L	Cuneus_L	Parietal_Inf_R	Occipital_Inf_R
**I_CBM**	Cuneus_R	Angular_R	Parietal_Sup_R
Cerebelum_Crus2_L	Occipital_Sup_L	Paracentral_Lobule_L	SupraMarginal_R
Cerebelum_Crus2_R	Occipital_Sup_R	Paracentral_Lobule_R	Paracentral_Lobule_R
Cerebelum_3_L	Angular_L	Caudate_R	Putamen_R
Cerebelum_4_5_R	Precuneus_L	Putamen_L	Pallidum_R

Cerebelum_7b_L	Precuneus_R	Putamen_R	Heschl_R
Cerebelum_7b_R	Paracentral_Lobule_L	Pallidum_R	Temporal_Pole_Sup_R
Cerebelum_8_L	Pallidum_L	Temporal_Sup_R	Temporal_Pole_Mid_R
Cerebelum_8_R	Thalamus_R	Vermis_6	**R_LIN**
Cerebelum_9_L	Temporal_Mid_L	Vermis_7	Frontal_Sup_Orb_R
Cerebelum_9_R	Cerebelum_4_5_L	Vermis_8	Rectus_R
Cerebelum_10_L	Cerebelum_6_L	Vermis_9	ParaHippocampal_R
Cerebelum_10_R	Temporal_Pole_Mid_L	Vermis_10	Lingual_R
Vermis_1_2	**L_CBM**	**R_CIG**	Fusiform_R
Vermis_3	Hippocampus_L	Cingulum_Mid_R	
Vermis_6	ParaHippocampal_L	Precuneus_R	
Vermis_7	Lingual_L	Caudate_L	
Vermis_8	Temporal_Sup_L	Thalamus_R	

Supplementary Table 2: Regions associated with each module obtained using Weighted Stochastic Block Model algorithm as depicted in figure 5. Here L and R indicates Left and Right hemispheres and color codes are corresponding to module colors in the figure.

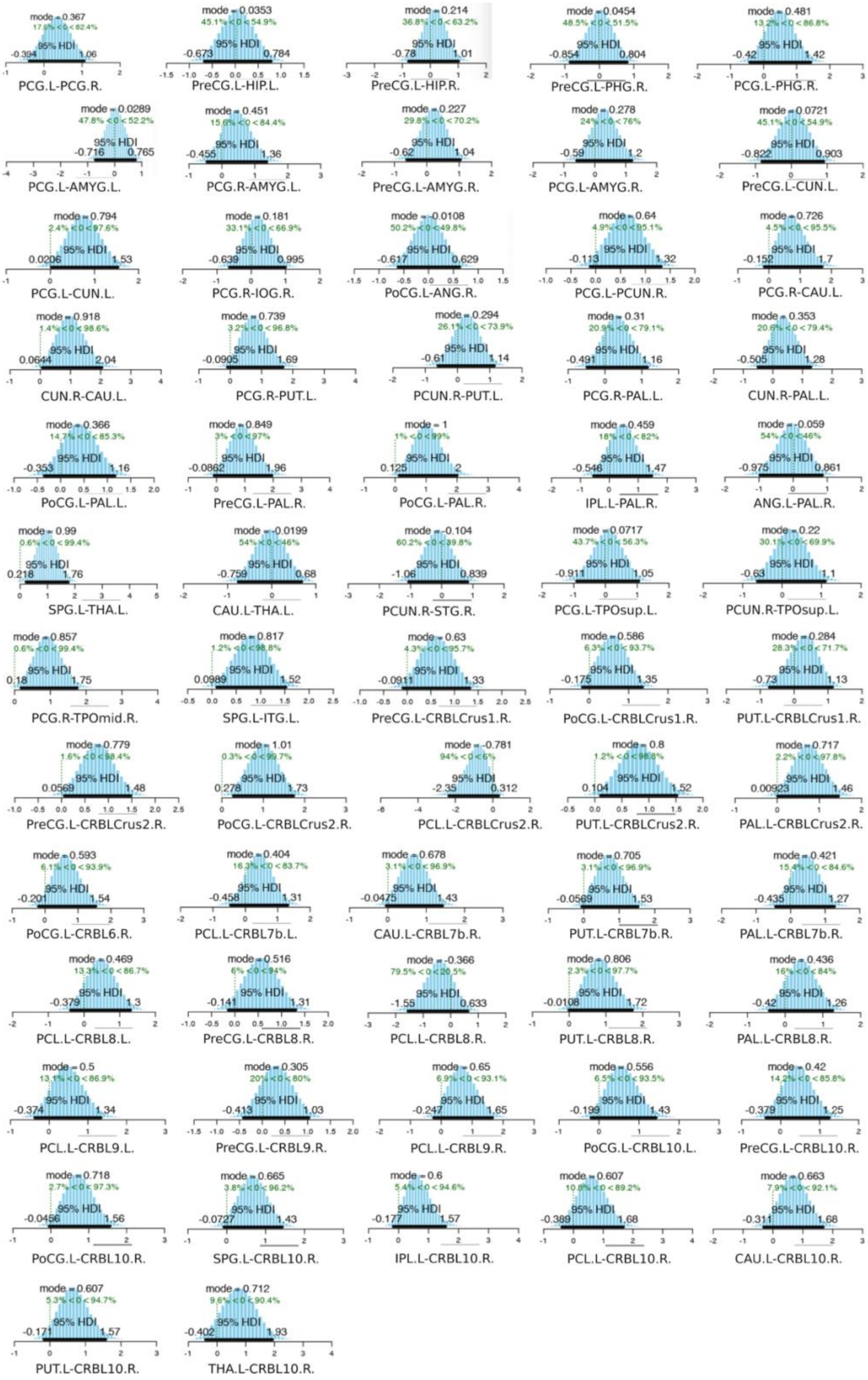

Supplementary Figure 1: Effect size distribution along with its modal value and high-density intervals for all connections obtained in network-based statistics (NBS) analysis.

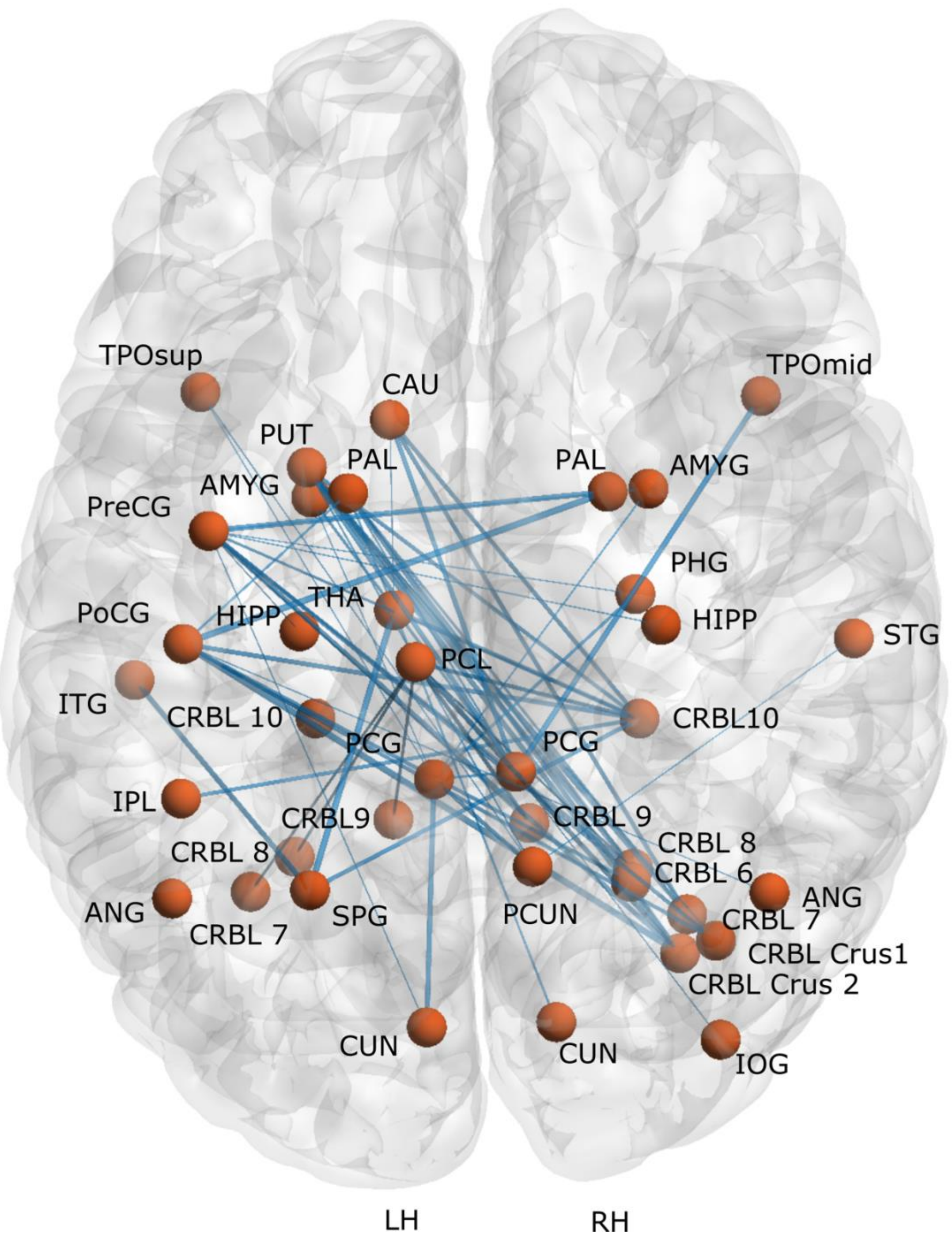

Supplementary Figure 2: The network with reduced connectivity for the adults who stutter compared to fluent speakers. Here the thickness of the edges (connections between regions) indicates the magnitude of the effect size for each connection as shown in supplementary figure 1.
